# Exploring vimentin's role in breast cancer via PICK1 alternative polyadenylation and the miR‐615‐3p‐PICK1 interaction

**DOI:** 10.1002/biof.2147

**Published:** 2025-01-09

**Authors:** Xinyan Jia, Lujing Shao, Hong Quan, Zhixian Zhong, Chunyan Dong

**Affiliations:** ^1^ College of Basic Medical Science Jinzhou Medical University Jinzhou Liaoning China; ^2^ Department of Oncology, Shanghai East Hospital, Tongji University School of Medicine Tongji University Shanghai People's Republic of China; ^3^ Department of Breast Surgery, Shanghai East Hospital, Tongji University School of Medicine Tongji University Shanghai People's Republic of China; ^4^ Ji'an Central People's Hospital (Ji'an Hospital of Shanghai East Hospital) Jiangxi Province China

**Keywords:** APA, miR‐615‐3p, PICK1, TNBC, VIM

## Abstract

Breast cancer continues to be a major health issue for women worldwide, with vimentin (VIM) identified as a crucial factor in its progression due to its role in cell migration and the epithelial‐to‐mesenchymal transition (EMT). This study focuses on elucidating VIM's regulatory mechanisms on the miR‐615‐3p/PICK1 axis. Utilizing the 4T1 breast cancer cell model, we first used RNA‐seq and proteomics to investigate the changes in the APA of PICK1 following VIM knockout (KO). These high‐throughput analyses aimed to uncover the underlying transcriptional and proteomic alterations associated with VIM's influence on breast cancer cells. RNA‐seq and proteomic profiling revealed significant APA in PICK1 following VIM KO, suggesting a novel mechanism by which VIM regulates breast cancer progression. Validation experiments confirmed that VIM KO affects the miR‐615‐3p‐PICK1 axis, with miR‐615‐3p's regulation of PICK1 being contingent upon the APA of PICK1. These findings highlight the complex interplay between VIM, miR‐615‐3p, and PICK1 in the regulation of breast cancer cell behavior. This study reveals that vimentin affects the miR‐615‐3p‐PICK1 axis through APA, revealing the key role of VIM in cancer progression. Opened up new avenues for targeted cancer therapy, with a focus on regulating the interaction between APA and miR‐615‐3p‐PICK1.

## Graphical abstract

Vimentin affects the miR‐615‐3p‐PICK1 axis via APA, highlighting its role in cancer progression in TNBC. The findings show the interplay between VIM, miR‐615‐3p, and PICK1 and open new avenues for targeted cancer therapy.

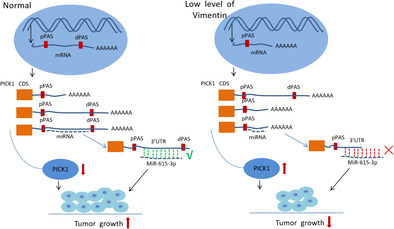

## 1 INTRODUCTION

Recent statistics underscore that breast cancer constitutes a significant public health challenge globally, with its incidence rates witnessing an upward trajectory over the past four decades. Given its prevalence and pronounced impact on both mortality and morbidity in women, the quest for a deeper understanding and more effective treatment modalities for breast cancer is far from over.^1^

Among the plethora of factors implicated in the progression of breast cancer, vimentin (VIM) has been identified as a pivotal element. As a type III intermediate filament, VIM plays an instrumental role in preserving cellular integrity, alongside facilitating critical processes such as cell migration, motility, and adhesion.^2^ Its overexpression is intricately associated with the epithelial‐to‐mesenchymal transition (EMT), a fundamental mechanism in the metastatic cascade of cancer.^3^ Notably, VIM's overexpression has been correlated with adverse clinical outcomes across a spectrum of cancers, including breast cancer.^4^ Recent scholarly contributions have posited that VIM's degradation could curtail the proliferation and metastasis of breast cancer cells, thereby underscoring its potential utility as both a biomarker and therapeutic target within the domain of breast oncology.^2^, ^5^, ^6^, ^7^ According to research reports, triple negative breast cancer has strong migration and invasion characteristics. It is not difficult to think of vimentin—one of the markers of epithelial mesenchymal transformation. Grasset et al.^8^ who conducted in‐depth research on the relationship between vimentin and triple negative breast cancer. They found that spontaneous TNBC tumors from genetic engineering mouse models (GEMM), xenografts from multiple patients, and archived patient samples showed a large number of mixed E/M cells in vivo, which led to invasion in vitro while expressing epithelial and mesenchymal characteristics. Similarly, Mehrdad Hashemi et al.^9^ reported that vimentin, an intermediate mesenchymal marker in triple negative breast cancer, promotes tumor invasion. For this reason, they sought lncRNAs microRNA as an entry point to interfere with the expression of vimentin and thus inhibit the metastasis of breast cancer. In addition, it was mentioned that vimentin inhibits focal adhesion‐related proteins to induce cell migration.^10^ On the contrary, in the absence of vimentin, cell migration will be inhibited by focal adhesion.^11^ When small interfering RNA was used to interfere with vimentin expression, the migration ability of breast cancer cells MDA‐MB‐231 was weakened, and the adhesion of MDA‐MB‐231 cells was also reduced. The vimentin also regulates the “cup‐shaped” structure of endothelial cells to promote cell migration. In conclusion, it confirmed that vimentin is involved in the invasion of breast cancer cells.^12^, ^13^

Recently, VIM has emerged as a critical player in the post‐transcriptional regulation of specific mRNAs, demonstrating the ability to interact with both the 3′‐untranslated region (3′‐UTR) and 5′‐untranslated region (5′‐UTR) of these molecules.^14^, ^15^ These findings suggest that VIM plays a pivotal role in the transcriptional regulation of mRNAs, potentially influencing the metastatic capability and progression of cancer cells.^14^ Vimentin can regulate the 3′UTR length of downstream mRNA through the APA mechanism, thereby exposing or hiding RNA binding proteins or miRNA binding sites and altering mRNA translation levels. To influence tumor progression in this way.^16^

Building on this premise, our investigation endeavors to elucidate the multifarious mechanisms through which VIM modulates breast cancer progression, with an emphasis on transcriptional regulation. By leveraging a comprehensive multiomics framework, integrating both transcriptomic and proteomic analyses, we aim to demystify the intricate nexus between VIM and breast cancer pathology. Such insights could potentially herald novel therapeutic interventions targeting this critical pathway.

## 2 METHODS

### 2.1 Cell culture

The 4T1 and MDA‐MB‐231 cell lines was acquired from Cyagen Biosciences (Guangzhou, China). 4T1 Cells and MDA‐MB‐231 cells were cultured in complete Dulbecco's Modified Eagle Medium (DMEM) and L15 medium separately, supplemented with 10% fetal bovine serum (FBS, catalog number 10099141C, Invitrogen, USA) and a penicillin–streptomycin‐glutamine mixture (catalog number 10378016, Invitrogen, USA). Cultures were maintained in a humidified atmosphere containing 5% CO_2_ at 37°C. The culture medium was refreshed every 3 days to ensure optimal growth conditions.

### 2.2 Lentivirus infection

Lentiviral vectors targeting miR‐615‐3p mimic and control lentiviral vectors were produced by GenePharma Co., Ltd. The 4T1 and MDA‐MB‐231 cells were infected with the lentivirus in the presence of polybrene (BL628A, Biosharp, China) for 12 h. Forty‐eight hours post‐infection, the infected 4T1 and MDA‐MB‐231 cells underwent antibiotic selection to ensure the integration of the viral construct. The target sequence for the miR‐615‐3p mimic is as follows: miR‐615‐3p mimic, 5′‐TCCGAGCCTGGGTCTCCCTCTT‐3′.

### 2.3 Quantitative real‐time PCR (qRT‐PCR)

Quantitative Real‐Time PCR (qRT‐PCR): Total RNA was isolated using the Vazyme Total RNA Isolation Kit (R711‐01, Vazyme, China) and cDNA was synthesized following the manufacturer's protocol using HiScript III RT SuperMix for qPCR (+gDNA wiper) (R323‐01, Vazyme, China). Real‐time PCR was performed using the MagicSYBR Mixture (CW3008, CWBIO, China). For miRNA detection, reverse transcription polymerase chain reaction (RT‐PCR) was conducted using the TaqMan MicroRNA Reverse Transcription kit (4366596, Invitrogen, USA). The TaqMan probe for hsa‐miR‐615 (Assay ID: 001960, Applied Biosystems, USA) was used to detect the specific microRNA, with U6 (Assay ID: 001973, Applied Biosystems) serving as the loading control. Quantitative real‐time PCR (qPCR) was conducted on a BioRad CFX96 Real‐Time PCR Detection System (BioRad, USA). Each reaction was performed in triplicate and the entire experimental process was repeated three times to ensure the reliability and consistency of the results. The relative expression levels were calculated using the $2^{-∆∆C_{t}}$ method.

### 2.4 Protein extraction and digestion

SDT(4%SDS, 100 mM Tris–HCl, pH 7.6) buffer was used for sample lysis and protein extraction. The amount of protein was quantified with the BCA Protein Assay Kit (Bio‐Rad, USA). 20 μg of protein for each sample were mixed with 5X loading buffer respectively and boiled for 5 min. The proteins were separated on 4%–20% SDS‐PAGE gel (constant voltage 180 V, 45 min). Protein bands were visualized by Coomassie Blue R‐250 staining. Protein digestion by trypsin was performed according to filter‐aided sample preparation (FASP) procedure described by Matthias Mann. The digest peptides of each sample were desalted on C18 Cartridges (Empore™ SPE Cartridges C18 (standard density), bed I.D. 7 mm, volume 3 mL, Sigma), concentrated by vacuum centrifugation and reconstituted in 40 μL of 0.1% (v/v) formic acid.

### 2.5 Western blot

Total protein was extracted using RIPA buffer. After quantification, equal amounts of protein samples were loaded and separated by 10% SDS‐PAGE, then transferred to a 0.45‐μm PVDF membrane. The membrane was blocked with 5% non‐fat milk for 2 h, followed by overnight incubation at 4°C with primary antibodies. The primary antibodies used were anti‐PICK1 (10983‐2‐AP, Proteintech, 1/1000 dilution) and anti‐GAPDH (ab8245, abcam, 1/5000 dilution). Subsequently, the membrane was incubated with secondary antibodies at room temperature for 1 h. Immunoblots were visualized using an ECL chemiluminescence detection kit (Beyotime, Shanghai) and observed with a Tanon 4600 system (Tanon Science and Technology Co., Ltd.).

### 2.6 Dual‐luciferase reporter assay

The coding sequences (CDS) of wild type (wt) or mutant (mut) PICK1 were cloned into the firefly luciferase expression vector pMIR‐REPORT (Sangon Biotech, Shanghai, China). The PICK1‐mut, PICK1‐wt, and Vector or miR‐615‐3p mimic were co‐transfected into the 4T1 cell line using Lipofectamine 3000 (Invitrogen, Carlsbad, CA, USA). The luciferase activity in the cells was normalized by co‐transfecting with a plasmid containing the full‐length Renilla luciferase gene (pTK‐Renilla) as a reporter (Genomeditech), and quantified 48 h after transfection using a Dual‐Luciferase Reporter Assay System (Promega).

### 2.7 5‐Ethynyl‐2′‐deoxyuridine (EdU) assay

The EdU incorporation was detected using the BeyoClick™ EdU Cell Proliferation Kit with Alexa Fluor 594 (Beyotime, Shanghai, China). After washing with PBS, the cells were incubated with the EdU solution for 2 h, followed by staining of the nuclei with DAPI solution. After washing, the samples were observed under an inverted microscope (Olympus).

### 2.8 Filter‐aided sample preparation (FASP digestion) procedure

The detergent DTT (with the final concentration of 10 mM) was added to each sample respectively and mixed at 600 rpm for 1.5 h (37°C). After the samples cooled to room temperature, IAA was added with the final concentration of 20 mM into the mixture to block reduced cysteine residues and the samples were incubated for 30 min in darkness. Next, the samples were transferred to the filters, respectively. The filters were washed with 100 μL UA buffer three times and then 100 μL 25 mM NH4HCO3 buffer twice. Finally, trypsin was added to the samples (the trypsin:protein (wt/wt) ratio was 1:50) and incubated at 37°C for 15–18 h (overnight), and the resulting peptides were collected as a filtrate. The peptides of each sample were desalted on C18 Cartridges (Empore™ SPE Cartridges C18 (standard density), bed I.D. 7 mm, volume 3 mL, Sigma), concentrated by vacuum centrifugation and reconstituted in 40 μL of 0.1% (v/v) formic acid. The peptide content was estimated by UV light spectral density at 280 nm using an extinctions coefficient of 1.1 of 0.1% (g/L) solution that was calculated on the basis of the frequency of tryptophan and tyrosine in vertebrate proteins.

### 2.9 LC–MS/MS analysis

We conducted peptide analysis using an LC–MS/MS setup, specifically a Q Exactive mass spectrometer from Thermo Scientific, connected to an Easy nLC system from Proxeon Biosystems (now part of Thermo Fisher Scientific). The peptides were first introduced onto a reverse phase trap column, Thermo Scientific Acclaim PepMap100 (100 μm by 2 cm, nanoViper C18), and then transferred to a C18‐reversed phase analytical column (Thermo Scientific Easy Column, 10 cm long, 75 μm inner diameter, with 3 μm resin) using a buffer solution of 0.1% Formic acid in water. This setup separated the peptides with a gradient mixture of 84% acetonitrile and 0.1% Formic acid at a 300 nL/min flow rate. Operating in positive ion mode, the mass spectrometer targeted the most abundant precursor ions for higher‐energy collisional dissociation (HCD) fragmentation, based on a data‐dependent top 20 method. We ensured precise measurement by setting the automatic gain control (AGC) target to 1e6, with a maximum injection time of 50 ms and a dynamic exclusion duration of 30.0 s. The system achieved a resolution of 60,000 at m/z 200 for survey scans and 15,000 at m/z 200 for HCD spectra, with an isolation width of 1.5 m/z. The normalized collision energy was set to 30 eV, and the underfill ratio was kept at 0.1%, ensuring that the system efficiently recognized and analyzed peptides.

### 2.10 Identification and quantitation of proteins

The MS raw data for each sample were combined and searched using the MaxQuant 1.6.14 software for identification and quantitation analysis.

### 2.11 Protein–protein interaction analysis

The protein–protein interaction (PPI) information of the studied proteins was retrieved from IntAct molecular interaction database (http://www.ebi.ac.uk/intact/) by their gene symbols or STRING software (http://string-db.org/). The results were downloaded in the XGMML format and imported into Cytoscape software (http://www.cytoscape.org/, version 3.2.1) to visualize and further analyze functional protein–protein interaction networks. Furthermore, the degree of each protein was calculated to evaluate the importance of the protein in the PPI network.

### 2.12 RNA extraction and RNA‐seq

Total RNA was extracted from tumor of both VIM‐KO and ctrl group. Nanodrop ND‐2000 (Thermo Scientific, USA) was used to detect the A260/A280 absorbance ratio of RNA samples. The Rins of RNA were determined by an Agilent Bioanalyzer 4150 system (Agilent Technologies, CA). Only qualified RNA can be used for library construction. Prepare paired terminal libraries and purify mRNA, followed by synthesizing cDNA using mRNA fragments as templates. Afterwards, the synthesized double stranded cDNA fragments were adapter linked to prepare a paired end library and subjected to PCR amplification. Purification and evaluation of PCR products (AMPure XP system), sequencing and 150 bp paired end reading on Illumina Novaseq 6000 (or MGISEQ‐T7). Subsequently, quality control and related analysis will be carried out.

### 2.13 NGS data analysis

The quality of raw sequencing data was examined by FastQC (v0.11.7). Contaminated adapter and low‐quality sequences were removed by using Trimmomatic (v0.38). Cleaned sequencing reads were then aligned to mm10 genome reference by STAR aligner (v2.5.2). Samtools (v1.3) was then used to remove unmapped reads and low‐quality alignments. Htseq‐count (v2.0.3) was used to calculate the raw counts for each transcript. The raw counts of different sample groups were compared by DESeq2 (v3.18) to identify the differentially expressed genes. For calling alternative polyadenylation (APA) events, DANPOS (v3.1.1) was used with default settings.

### 2.14 Functional enrichment analysis

To assess the biological functions, the computational web server g:Profiler^17^ (https://biit.cs.ut.ee/gprofiler/gost) was utilized to conduct Gene Ontology (GO) and Kyoto Encyclopedia of Genes and Genomes (KEGG) pathway analyses for differentially expressed genes. The resulting GO and KEGG pathway terms were ranked according to the *p* value and visualized using R software (v4.1.2).

### 2.15 APA analysis

An in‐depth analysis and prediction of APA were conducted on RNA‐Seq data based on Dapars software (following the PDUI scoring principle). The closer the PDUI score is to 1, the longer the 3′UTR of the transcript. Conversely, the closer the PDUI score is to 0, the shorter the 3′UTR of the transcript.

### 2.16 CCK8 cell proliferation assay

Inoculate the processed cells into a 96‐well plate, add the CCK8 detection reagent at different time points, and incubate in the dark for several hours in the incubator. Finally, detect the OD values of the cells at a wavelength of 450 nm.

### 2.17 Cell cloning experiment

Inoculate the processed cells into a six‐well plate, ensuring that the cell density is not too high. Regularly change the culture medium, and perform crystal violet staining after ~14 days.

### 2.18 Wound healing assay

Inoculate the processed cells into a six‐well plate, and when the cell confluence reaches 70%, perform vertical streaking. Then, thoroughly wash the cells with PBS, add serum‐free culture medium, and observe under a microscope at different time points, taking pictures for record.

## 3 RESULTS

### 3.1 RNA‐seq reveals the fundamental transcription alternation of VIM KO breast cancer cells

**FIGURE 1:**
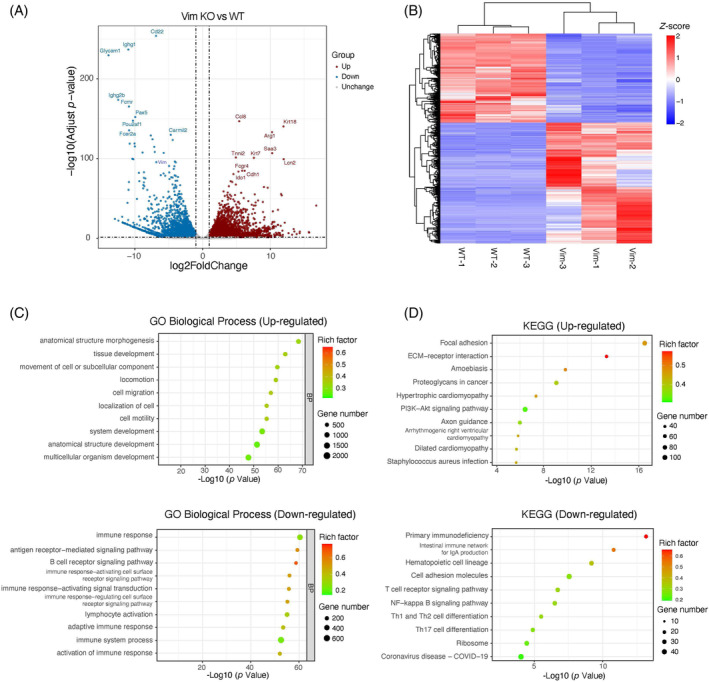
Transcription profiling of Vim KO and WT breast cancer cells. (A) The volcano plot showing the significant differentially expressed genes between Vim KO and WT breast cancer cells. (B) Heatmap visualization of the DEGs. (C) Gene Ontology analysis of up and downregulated DEGs. (D) KEGG pathway enrichment of up and downregulated DEGs.

We initially conducted an analysis of the relationship between VIM expression and the prognosis of breast cancer using the website https://bcgenex.ico.unicancer.fr/BC-GEM/GEM-Requete.php?mode=2. It was found that the lower the expression of VIM, the longer the survival period of breast cancer patients (Figure S1), indicating a negative correlation between the two. To investigate the roles of VIM in transcription regulation, we performed RNA‐seq experiments on both VIM‐KO and WT breast cancer cell line—4T1. Consistent to the fundamental functions of VIM, VIM‐KO induced massive transcription alternations in 4T1, including 4245 significant upregulated differentially expressed genes (DEGs) and 3147 significant downregulated DEGs (Figure 1A,B). DEGs was defined by log2FoldChange >1 and adjusted *p* value <0.05. Based on Gene Ontology analysis, upregulated DEGs were enriched to anatomical structure morphogenesis, tissue development, movement of cell or subcellular component, cell migration, and cell motility. The downregulated DEGs were enriched to immune‐related terms, such as immune response, antigen receptor‐mediated signaling pathway, and B cell receptor signaling pathway (Figure 1C). Similarly, based on KEGG pathway enrichment analysis, upregulated DEGs were enriched to focal adhesion, ECM‐receptor interaction, proteoglycans in cancer, and PI3K‐Akt signaling pathway. The downregulated were enriched to primary immunodeficiency, cell adhesion molecules, and T cell receptor signaling pathway (Figure 1D). These results demonstrate not only the crucial roles of VIM in maintaining the cell integrity, regulating cell migration, and cancer cell proliferation but also its involvement in immune responses regulation, suggesting VIM contributes to the development, proliferation, and migration of cancer cells through various mechanisms.

### 3.2 Proteomic profiling of VIM KO breast cancer cells

**FIGURE 2:**
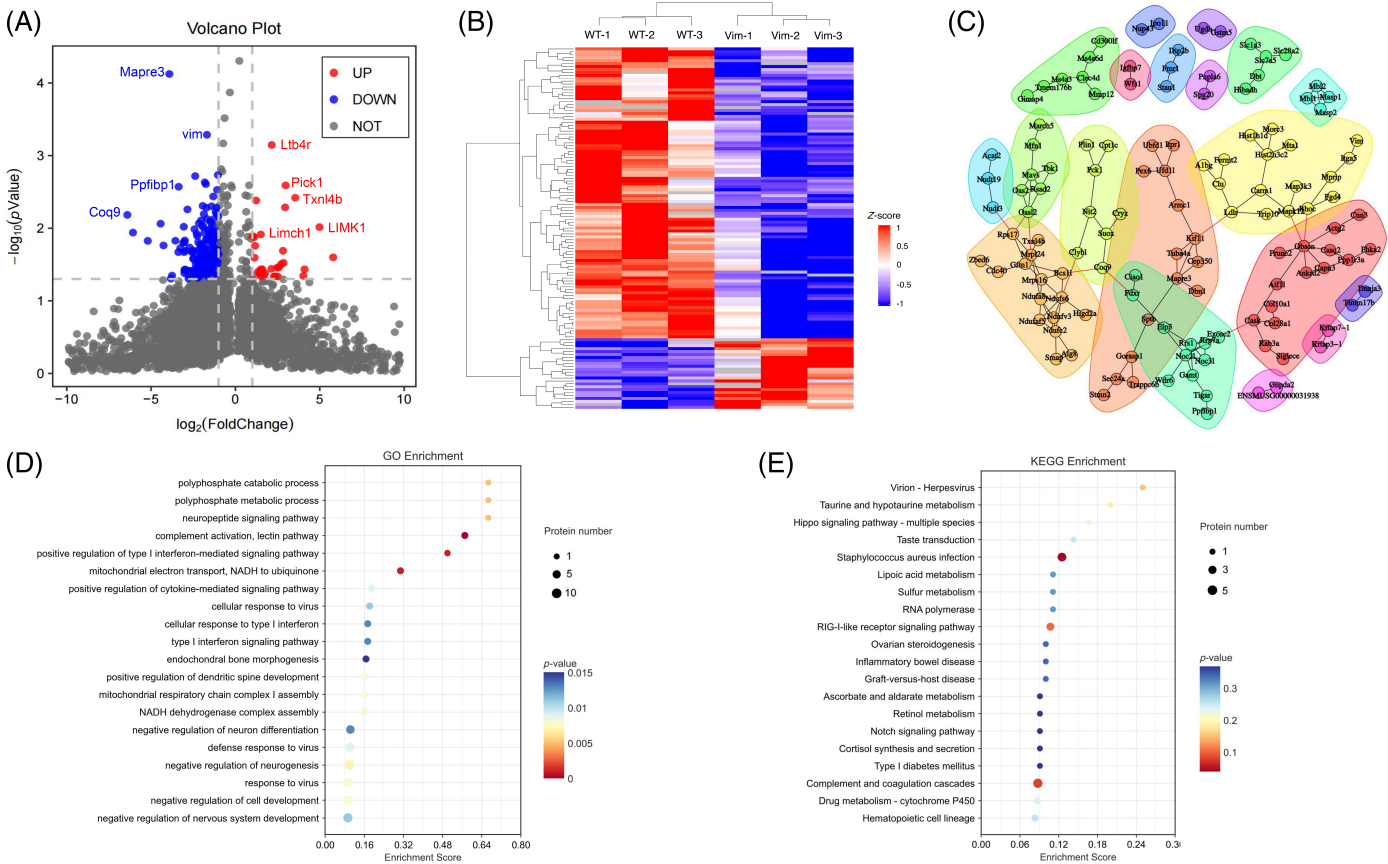
Proteomic profiling of Vim KO and WT breast cancer cells. (A) The volcano plot showing the significant DEGs between Vim KO and WT breast cancer cells at protein level. (B) Heatmap visualization of DEGs at protein level. (C) Clusters of protein and protein interactions. Gene Ontology (D) and KEGG pathway (E) analysis of DEGs at protein level.

To further study the functions of VIM in protein level, we performed LC–MS/MS analysis to profile the protein levels of both WT and VIM KO breast cancer cells (4T1). In VIM KO group, the VIM protein level was reduced to 6.5% compared to WT breast cancer cells, confirming the efficient of VIM knockout in VIM KO cells. Compared to WT group, we identified 24 upregulated proteins and 99 downregulated proteins (Figure 2A,B). The top upregulated genes include Limch1, Ltb4r, LIMK1, and Txnl4b, Pick1, which are involved in cell migration, immune response and cell cycle progression, respectively. Top downregulated genes include Mapre3 and Ppfibp1, Vim, Coq9, which are involved in regulating cell adhesion and migration. Based on the protein–protein interaction analysis performed on DEGs, we observed three major clusters. Genes of Cluster 1 is enriched to epithelial mesenchymal transition pathway, including Vim, Itga5, and Fermt2. Genes of Cluster 2 is enriched to extracellular matrix organization, including Col28a1, Cask, Col10a1, and Capn3. Genes of Cluster 3 is mainly related to microtubule construction, with Mapre3 and Kif11 being the center node of this cluster (Figure 2C). Additionally, we also performed gene ontology analysis and KEGG pathway enrichment analysis on DEGs at protein level and observed a significant enrichment to immune and inflammation‐related terms or pathways, such as complement activation, positive regulation of type I interferon‐mediated signaling pathway and complement and coagulation cascades. DEGs at protein level are also enriched to metabolism‐related terms or pathways, such as polyphosphate catabolic process, taurine and hypotaurine metabolism, lipoic acid metabolism, and sulfur metabolism, suggesting Vim KO has an impact on the energy supply that are required to tumor growth (Figure 2D,E).

### 3.3 Vim regulates the EMT of breast cancer cells through APA

**FIGURE 3:**
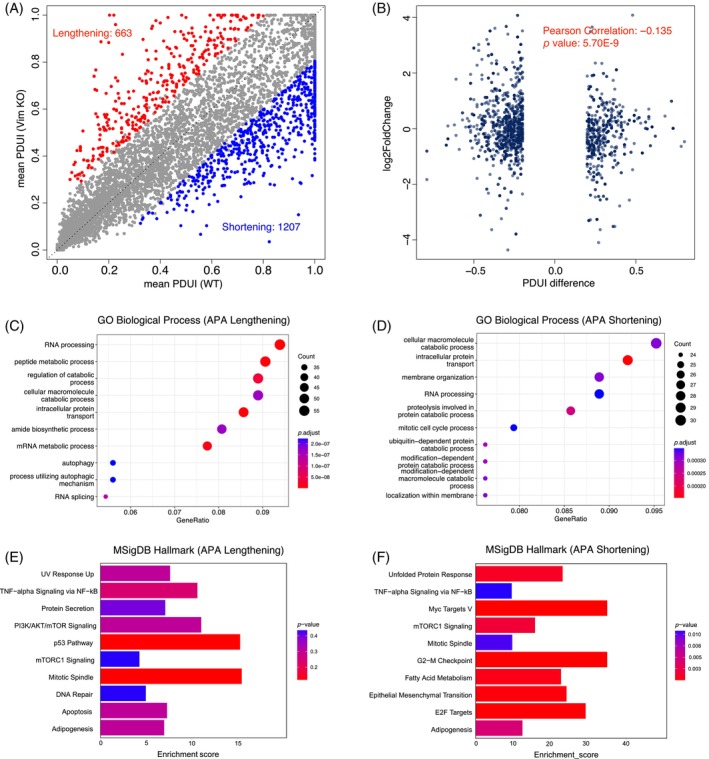
Vim regulates transcription alternation through alternative polyadenylation in breast cancer cells. (A) The scatter plot demonstrating the significant lengthening and shortening genes identified in Vim KO breast cancer cells by DANPOS. (B) The scatter plot showing the correlation between PDUI value and gene expression level. Gene ontology analysis of the APA lengthening (C) and shortening genes (D). The MSigDB Hallmark enrichment of the APA lengthening (E) and shortening genes (F).

APA is one of the major mechanisms of posttranscriptional regulation and has been demonstrated to be involved in cancer development and progression.^18^ To investigate whether Vim regulates gene expression through this mechanism, we compared Vim KO and WT breast cells and identified 1207 significant shortening transcripts and 663 lengthening transcripts (Figure 3A). Consistent to previous report that transcript shortening is related to upregulation of gene expression,^19^ we observed significant negative correlation (Pearson correlation: −0.135; *p* value: 5.70E‐9) between gene expression and the percentage of distal poly(A) site usage index (PDUI) value (Figure 3B). Based on the gene ontology analysis, the lengthening transcripts are enriched to RNA processing, peptide metabolic process, regulation of catabolic process and autophagy. The shortening transcripts are enriched to cellular macromolecule catabolic process, intracellular protein transport, membrane organization and mitotic cell cycle process (Figure 3C,D). We further investigated the enrichment of lengthening and shortening transcripts to the MSigDB Hallmark pathway. The lengthening transcripts are enriched to p53 pathway, mitotic spindle and PI3K/AKT/mTOR signaling while the shortening transcripts are enriched to G2‐M checkpoint, Myc Targets V and epithelial mesenchymal transition (Figure 3E,F). These results indicates that Vim KO caused fundamental transcription alternations are partially through APA's regulation on RNA processing and metabolism. Vim KO also influence crucial pathways related to cancer survival, proliferation, and malignancy through APA mechanism.^20^

**FIGURE 4:**
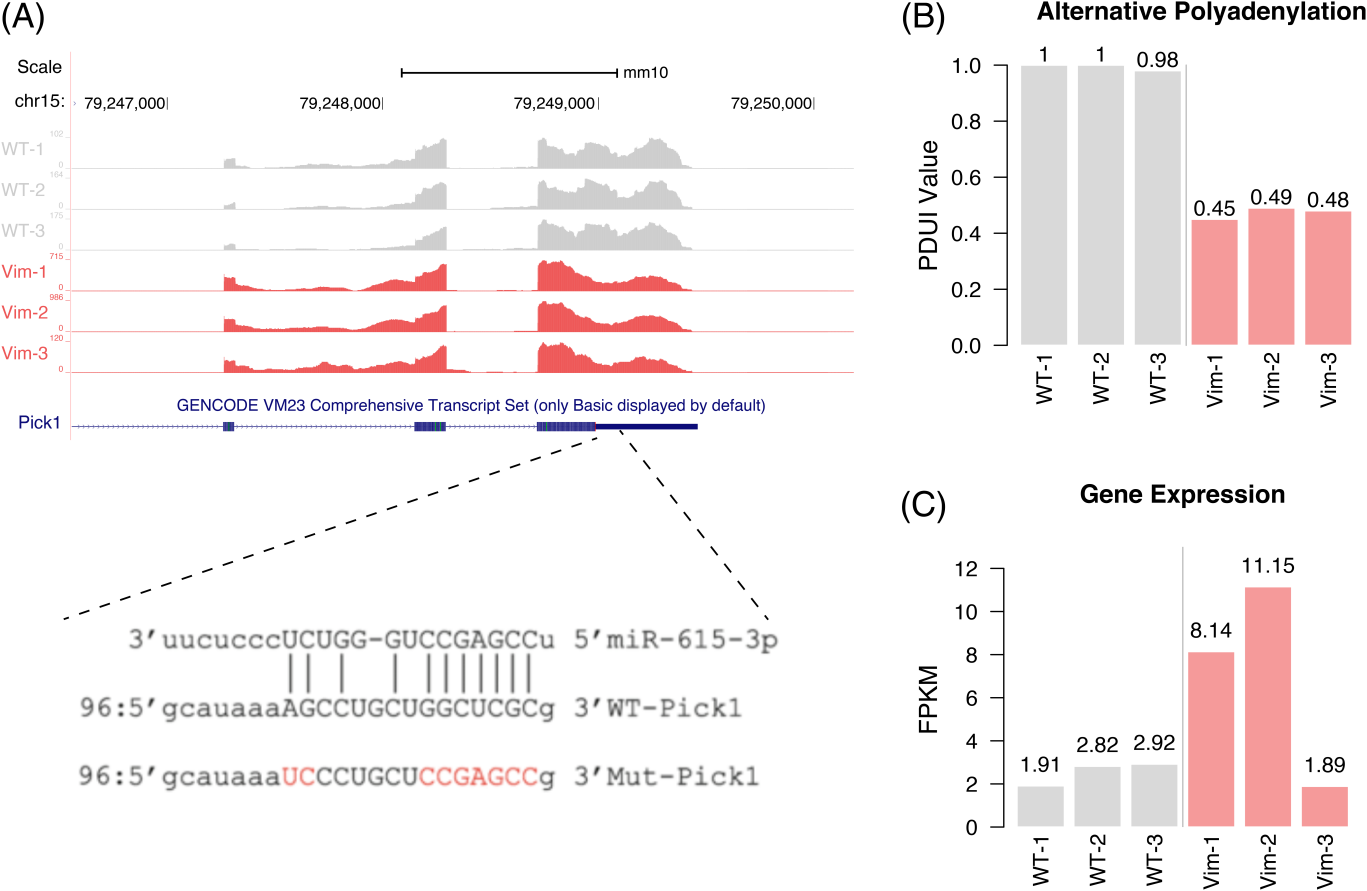
Vim KO induced the usage of proximal PAS of Pick1. (A) The APA shortening event identified in the 3′UTR region of Pick1 in Vim KO breast cancer cells. Bar plot showing the decreased PDUI value after Vim KO (B) and the corresponding upregulated gene expression level (C).

Among the shortening transcripts in Vim KO breast cancer cells, the APA event observed in the 3′‐UTR of Pick1 gene is one of the most significant events and associated with the EMT process. Pick1 was reported to be the negative regulator of TGF‐beta signaling and inhibit the EMT process and cancer metastasis.^21^ Based on DANPOS results, the distal polyadenylation site (PAS) of Pick1 is mainly used in WT breast cancer cells while the usage of proximal PAS is significantly increased in Vim KO breast cancer cells. Given previous studies, the 3′‐UTR of Pick1 contains the target site of miR‐615‐3p, which suppresses the Pick1/TGF‐beta signaling axis and promotes the EMT and breast cancer metastasis^22^ (Figure 4A). The knockout of Vim induced the increasing usage of proximal PAS and the abrogates of the miR‐615‐3p target sites, therefore causing the upregulation of Pick1 at transcription level and the inhibition of EMT process (Figure 4B,C). These results indicate that after KO‐VIM, under the regulation of APA mechanism, the 3′UTR of downstream PICK1 is shortened, resulting in a decrease in its expression level translated into protein. Therefore, in this study, vimentin showed a negative correlation with downstream genes through APA mechanism.

### 3.4 miR‐615‐3p negatively regulates PICK1 to promote breast cancer progression, with its effectiveness depending on the presence or absence of VIM knock‐out

**FIGURE 5:**
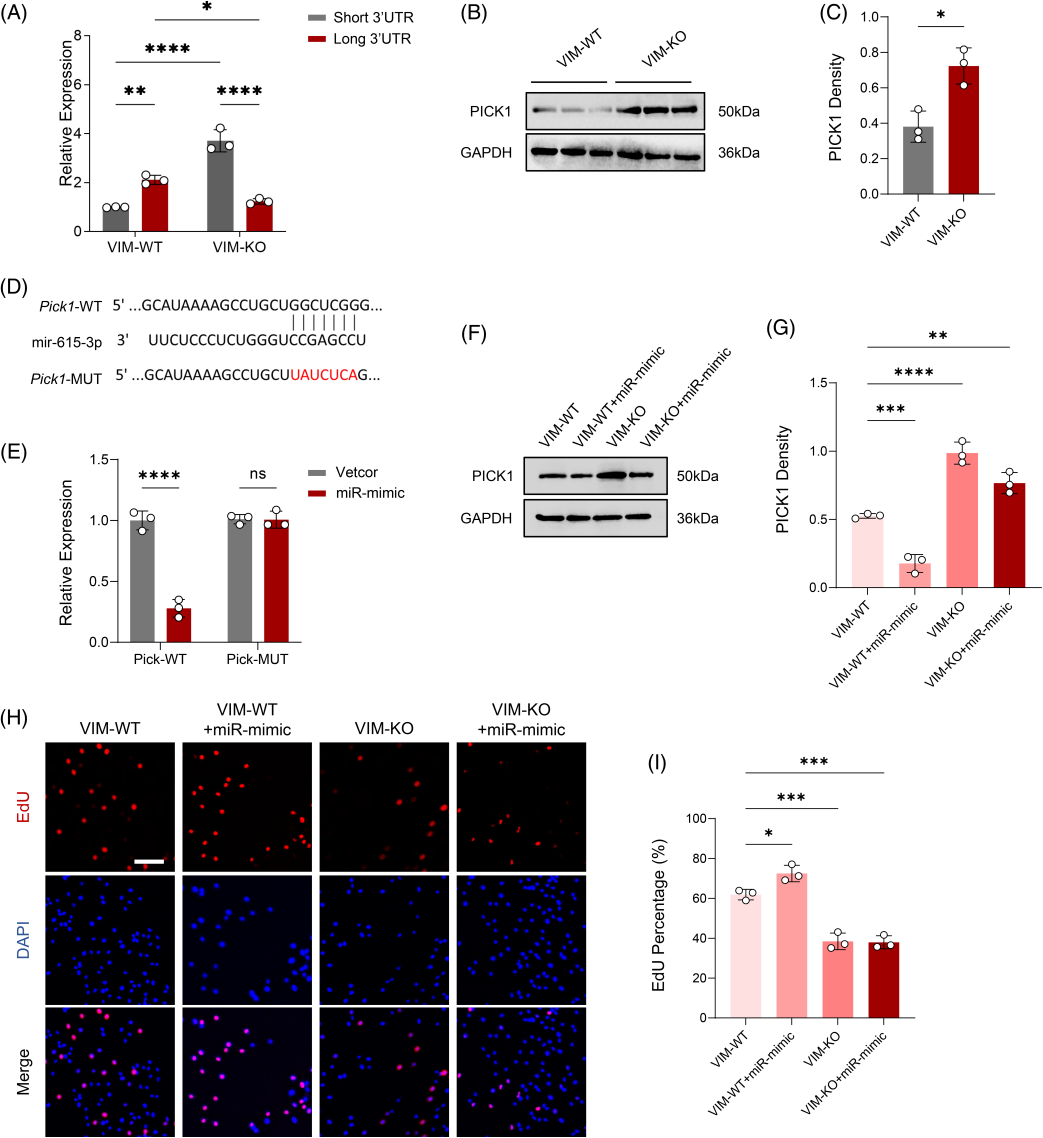
miR‐615‐3p negatively regulates PICK1 to promote the progression of breast cancer. (A) qRT‐PCR analysis of the transcription levels of the short and long 3′UTR of PICK1 in VIM‐WT and VIM‐KO 4T1 cells, with relative quantification. (*n* = 3) (B, C) Western Blot analysis of the protein levels of PICK1 in VIM‐WT and VIM‐KO 4T1 cells, with relative quantification. (*n* = 3) (D) The binding region of wild‐type PICK1 with miR‐615‐3p and the sequence information of mutant PICK1. (E) Dual‐luciferase assay demonstrates the interaction between PICK1 and miR‐615‐3p. (*n* = 3) (F, G) Western Blot analysis of the changes in PICK1 protein levels after transfection of miR‐615‐3p mimic in VIM‐WT and VIM‐KO 4T1 cells, with relative quantification. (*n* = 3) (H, I) EdU staining to assess the viability changes of the 4T1 cell line after transfection of miR‐615‐3p mimic in VIM‐WT and VIM‐KO 4T1 cells, with quantitative analysis of the proportion of EdU‐positive cells. (*n* = 3), Scale bar = 50 μm.

We next focused on elucidating the influence of miR‐615‐3p on breast cancer progression through its interaction with PICK1. Initial analysis using qRT‐PCR demonstrated significant transcriptional variations between the short and long 3′‐UTRs of PICK1 in both VIM‐WT and VIM‐KO 4T1 cells, suggesting a differential regulatory mechanism that could be attributable to APA influenced by the presence or absence of VIM (Figure 5A). Protein levels of PICK1, as determined by Western Blot, further confirmed these transcriptional differences, revealing a nuanced layer of regulation potentially driven by miR‐615‐3p's interaction with PICK1, thereby modulating the protein's expression (Figure 5B,C).

The sequence specificity of this interaction was highlighted through the delineation of the binding region between wild‐type PICK1 and miR‐615‐3p, alongside the introduction of mutations within PICK1 that presumably disrupt this interaction. This aspect of the study underscores the targeted nature of miR‐615‐3p's regulatory role (Figure 5D). The functionality of this interaction was validated via dual‐luciferase reporter assays, which unequivocally demonstrated miR‐615‐3p's engagement with PICK1, offering a molecular basis for the post‐transcriptional regulation observed (Figure 5E).

Subsequent analyses focused on the impact of miR‐615‐3p mimic transfection in both VIM‐WT and VIM‐KO cells. Western Blot tests after transfection showed clear changes in the levels of PICK1 protein, highlighting miR‐615‐3p's key role in controlling PICK1 expression. However, when VIM was knocked out, the miR‐mimic did not lower the protein expression, suggesting that the effect of miR‐615‐3p on PICK1 depends on whether VIM is present or not (Figure 5F,G). EdU proliferation assays further illuminated the functional consequences of this regulatory pathway, showing that miR‐615‐3p's modulation of PICK1 expression significantly affects the proliferative capacity of 4T1 cells in both VIM‐WT and VIM‐KO contexts (Figure 5H,I). This series of experiments provides strong evidence that miR‐615‐3p can effectively downregulate PICK1, while the regulatory region of PICK1 by miRNA is influenced by the presence or absence of VIM. This regulatory interaction plays a crucial role in advancing breast cancer progression, likely by causing changes in the cellular environment that favor tumor growth and spread.

### 3.5 In vitro experiments have confirmed that the binding of MiR‐615‐3p to PICK1 promotes TNBC proliferation mainly through vimentin

**FIGURE 6:**
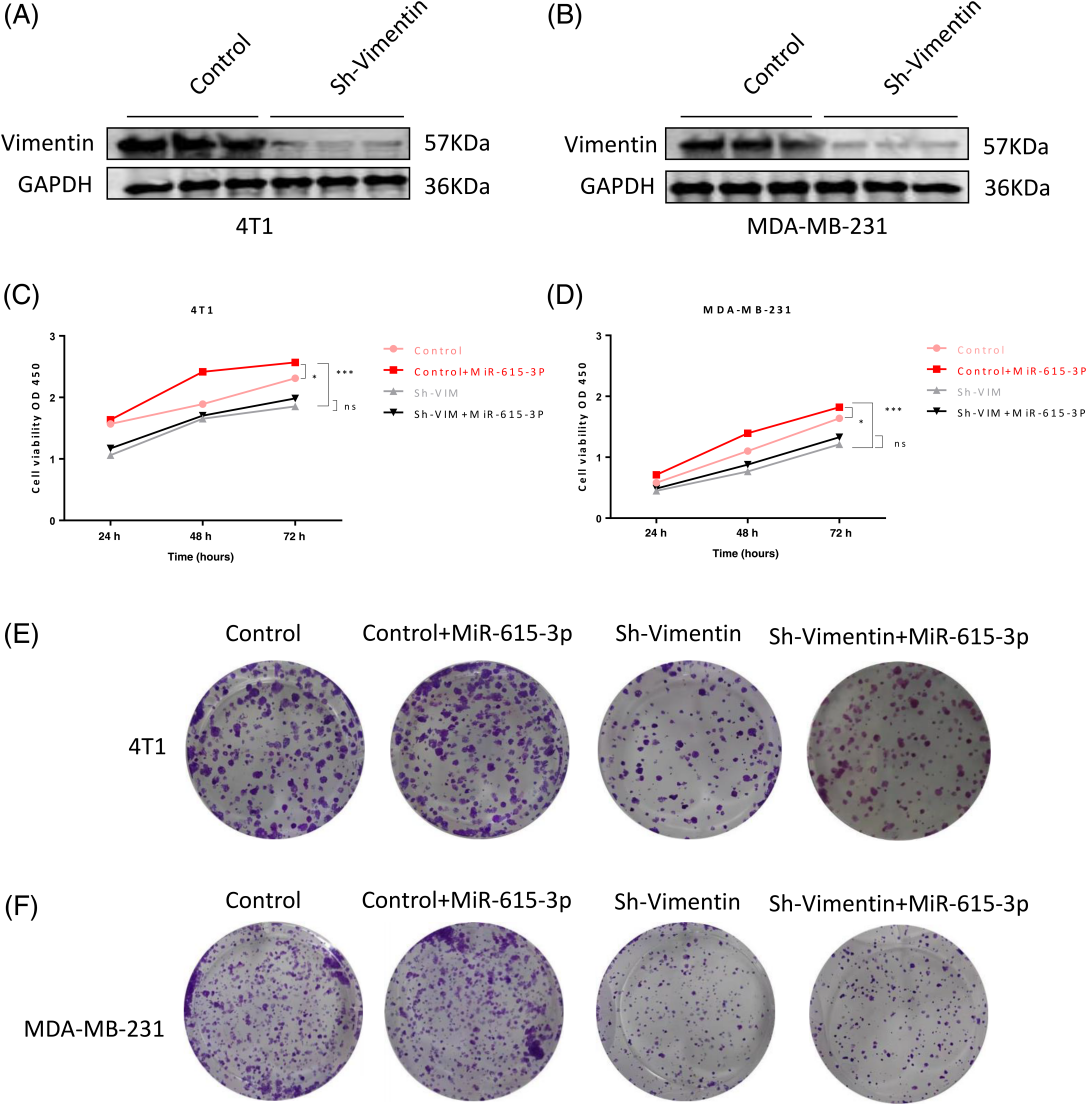
In vitro experiments have confirmed that the binding of MiR‐615‐3p to PICK1 promotes TNBC proliferation mainly through Vimentin. (A, B) Vimentin knockdown was validated in 4T1 and MDA‐MB‐231 cells. (C, D) The effect of miR‐615‐3p mimetics transfected with Control and Sh vimentin on the proliferation of 4T1 and MDA‐MB‐231 cells. (E, F) The effect of transfection of miR‐615‐3p mimetics based on Control and Sh vimentin on clone formation in 4T1 and MDA‐MB‐231 cells.

**FIGURE 7:**
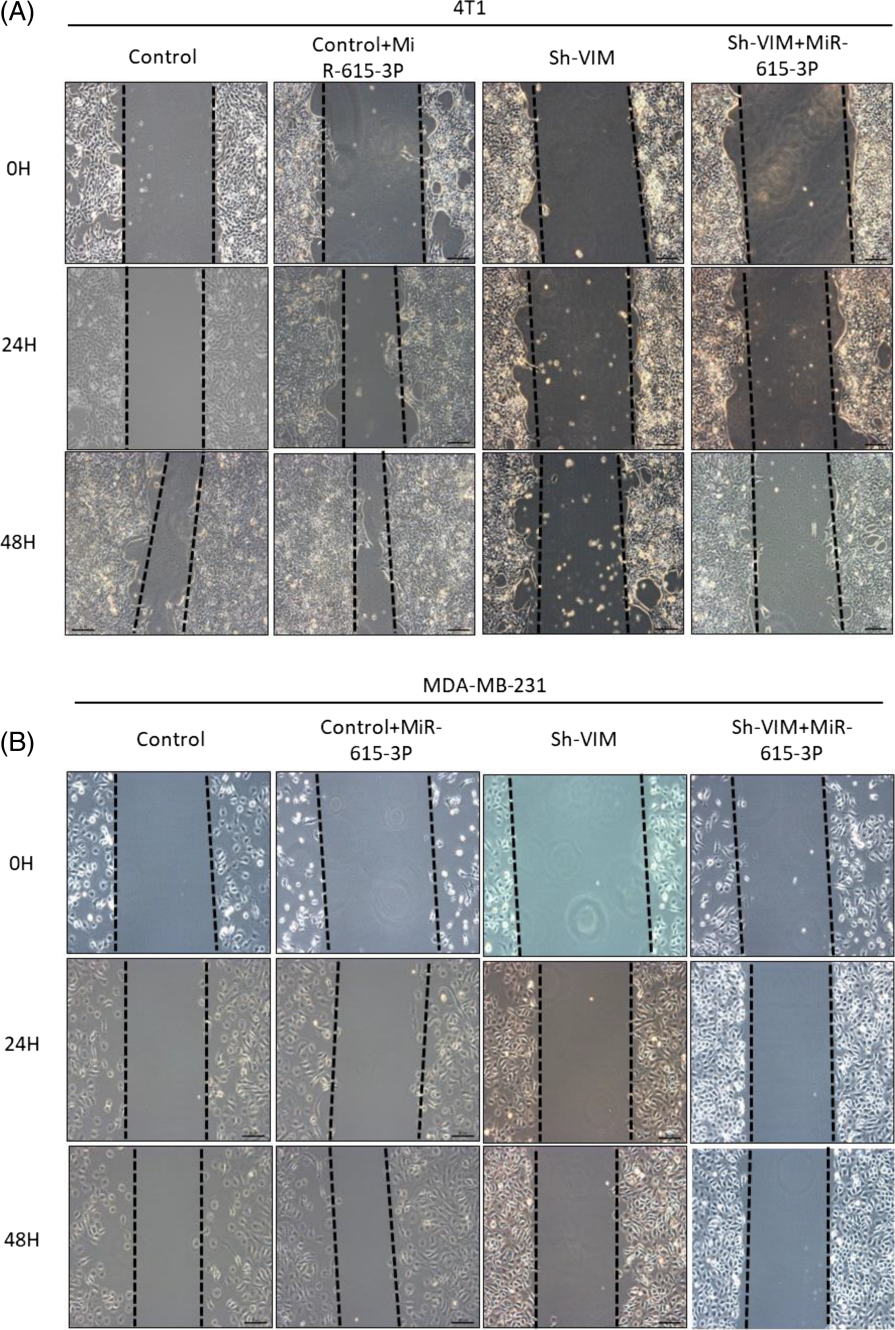
In vitro experiments have confirmed that the binding of MiR‐615‐3p to PICK1 mainly promotes TNBC migration through vimentin. (A, B) The effect of miR‐615‐3p mimetics transfected with Sh NC and Sh vimentin on scratch migration in 4T1 and MDA‐MB‐231 cells.

Through the above research, we have learned that whether MiR‐615‐3p can bind to PICK1 mainly depends on the presence of vimentin. To further verify this hypothesis, we selected 4T1 and MDA‐MB‐231 three negative breast cancer cells for verification. Firstly, knock down vimentin on the cells (Figure 6A,B). Next, the experimental groups were divided into four groups: Control group, Control+MiR‐615‐3p group, Sh vimentin group, and Sh vimentin+MiR‐615‐3p group. And CCK8 experiment and plate cloning experiment were conducted to detect its effect on TNBC cell proliferation. The CCK8 experiment results showed that the Sh vimentin group had the strongest ability to inhibit cell proliferation, followed by the Sh vimentin+MiR‐615‐3p group, Control group, and Control+MiR‐615‐3p group (Figure 6C,D). The same conclusion was obtained from the plate cloning experiment (Figure 6E,F). And in Wound‐Healing, the Sh vimentin group had the strongest ability to inhibit cell migration, followed by the Sh vimentin+MiR‐615‐3p group, control group, and control+MiR‐615‐3p group (Figure 7A,B). In addition, we found that miR‐615‐3P simulated the enhancement of TNBC cell proliferation and metastasis in control cells, but did not show significant proliferation and metastasis in Sh vimentin cells. These results further indicate that the regulation of TNBC proliferation by MiR‐615‐3p binding to PICK1 relies on the presence of vimentin.

## 4 DISCUSSION

Our study revealed that VIM‐KO significantly alters both the transcriptome and proteome of breast cancer cells, specifically increasing the expression of short 3′‐UTRs. These finding highlights VIM's role in transcriptional regulation, with a particular emphasis on the crucial function of alternative splicing. Moreover, our research demonstrated that miR‐615‐3p binds more readily to the longer 3′‐UTRs of *PICK1* mRNA. However, VIM‐KO leads to shorter 3′‐UTRs, thereby affecting miR‐615‐3p's binding and consequently upregulating PICK1 expression. This upregulation inhibits cell proliferation in breast cancer.

VIM is a type III intermediate filament protein essential for maintaining cellular integrity and stability. Ubiquitously expressed in mesenchymal cells, VIM is a key marker of EMT and interacts with various signaling pathways, influencing a wide range of cellular functions.^6^, ^8^, ^23^ Although direct references to VIM's specific impact on the 3′‐UTR or 5′‐UTR regions of mRNAs are relatively sparse, its involvement in transcriptional regulation is intriguing. For example, VIM binds to the 5′‐UTR stem‐loop domain of collagen mRNAs, regulating collagen synthesis, and interacts with the 3′ UTR of certain mRNAs, such as alkaline phosphatase mRNA in human primary osteoblasts and tissue factor mRNA in human breast cancer cells, stabilizing them and thus influencing their expression levels.^24^ Specifically, in breast cancer, VIM has been shown to prevent miR‐dependent negative regulation of tissue factor mRNA by interacting with its 3′‐UTR during EMTs of circulating tumor cells, promoting coagulant activity associated with early metastasis.^14^

Weibiao Kang et al.^25^ found significant changes in the length of downstream genes 3′UTR after CPSF1 knockdown in gastric cancer through transcriptome sequencing. Among them, NSDHL is positively regulated by CPSF1 and promotes the progression of gastric cancer. ShiXing Long et al.^26^ found through global APA site analysis that knocking out CPSF6 can induce extensive 3′UTR gene shortening in gastric cancer cells. CPSF6 negatively regulates VHL expression through APA and short 3′UTR subtypes, enhances GC cell apoptosis, and inhibits cell growth. Zhang Shirong et al. found in their study^27^ that polyadenylation (APA) site transitions in 3′UTR are common in non‐small cell lung cancer, and CSTF2 may serve as an oncogene to regulate the 3′UTR length of cancer‐related genes in non‐small cell lung cancer. Huang Jingjing et al.'s study^28^ showed that CFIm25 plays an important role in lung cancer cell proliferation by regulating the APA of oncogenes (including IGF1R) and promoting their protein expression.

In our study, we discovered that VIM‐KO does not directly regulate PICK1 expression but instead modulates it by affecting the length of the 3′‐UTR, suggesting that the mechanism of APA is involved in regulating the length of *PICK1* mRNA 3′‐UTR in VIM‐KO cells. APA is a critical mechanism in gene expression, producing mRNA isoforms with differing 3′‐UTR lengths, thereby affecting mRNA stability, translation efficiency, and cellular localization.^29^, ^30^, ^31^ This process is regulated co‐ and post‐transcriptionally, influenced by interactions between polyadenylation factors and RNA‐binding proteins, and affected by the cellular environment.^32^ In our research and obtained results, it is assumed that vimentin is mostly positively correlated with the length of the downstream target gene 3′UTR. Because when vimentin is knocked down, PICK1 shows a significant 3′UTR shortening, which may be related to vimentin as a mesenchymal marker. But the specific relationship is still unclear and requires further exploration. Although the connection between VIM and APA has not been explored, it presents a promising area for future research. Moreover, the modulation of PICK1 expression by VIM through altering the 3′ UTR length hints at a potential role for APA in cancer progression, similar to the application of comprehensive genomic analyses in the interpretation and staging of lung cancer,^33^, ^34^ suggesting that the length of the 3′ UTR might be a significant factor in the staging or prognosis of breast cancer.

MicroRNAs (miRNAs) are key regulators of gene expression at the transcriptional level, often binding to complementary sequences within the 3′‐UTR of target mRNAs to mediate their degradation or inhibit their translation.^35^, ^36^, ^37^ Lei et al.^22^ elucidated the role of miR‐615‐3p in promoting the EMT and metastasis of breast cancer by targeting the PICK1/TGFBRI axis. According to their study, miR‐615‐3p facilitates breast cancer metastasis by directly targeting the 3′‐UTR of *PICK1* mRNA, leading to reduced PICK1 expression, which in turn influences TGF‐β signaling and its downstream effects on cell migration and invasion. We discovered an upregulation of PICK1 in VIM‐KO breast cancer cells, suggesting a novel regulatory mechanism whereby VIM may control breast cancer progression through the miR‐615‐3p/PICK1 axis.

Currently, vimentin has been extensively studied in various tumors (including breast cancer). As a post‐transcriptional regulatory mechanism, alternative polyadenylation (APA) is relatively novel. Although there are literature reports on the progress of APA in regulating various tumors, and APA‐related factors have been used as the starting point to conduct research on the mechanism of APA and search for downstream genes, no one has explored the regulatory mechanism of APA using vimentin as a starting point. To this end, we regulated the downstream target gene PICK1 through the APA mechanism of vimentin, thereby affecting the miR‐615‐3p/PICK1 axis, inhibiting cell proliferation and reducing metastasis. These findings highlight previously unrecognized pathways by which vimentin affects cancer progression, providing promising directions for targeted therapy strategies targeting the miR‐615‐3p/PICK1 axis in cancer treatment.

## AUTHOR CONTRIBUTIONS

Xinyan Jia, Lujing Shao, Hong Quan, Zhixian Zhong and Chunyan Dong conceived the project and participated in the study design and interpretation of the results. Xinyan Jia and Lujing Shao wrote the manuscript. Xinyan Jia and Chunyan Dong participated in the study design and helped draft the manuscript. Hong Quan and Lujing Shao participated in data interpretation and provided a critical review of the manuscript. Zhixian Zhong mainly participated in the revision and finalization of the article.

## FUNDING INFORMATION

This work was supported by the National Natural Science Foundation of China (82073387), Shanghai Pudong New District Health and Family Planning Commission Industry Specialization (PW2021E‐05), Shanghai Pudong New Area Health and Family Planning Commission Leading Talent Training Program (Grant number: PWRI2019‐07) and Jiangxi Provincial Key Research and Development Program (S2021ZPYFE0649).

## CONFLICT OF INTEREST STATEMENT

All the authors have declared that no competing interests exist.

## CONSENT FOR PUBLICATION

All authors reviewed and approved the manuscript.

## Supporting information


Figure S1

## Data Availability

The datasets generated and/or analyzed during the current study are available from the corresponding author upon reasonable request.
