# Supplementary figures and images for: Exploring vimentin's role in breast cancer via PICK1 alternative polyadenylation and the miR‐615‐3p‐PICK1 interaction
Source: Biofactors. 2025 Jan 9;51(1):e2147. doi: 10.1002/biof.2147 (PMC11712540; doi:10.1002/biof.2147)

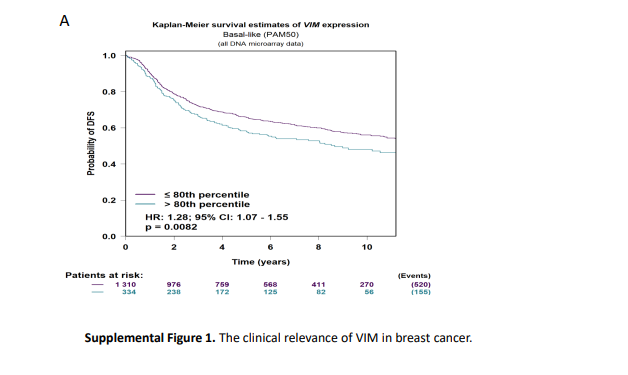

Supplement: Supplementary file 1 — Figure S1 [file BIOF-51-0-s001.png]
